# INTEREST GROUP SESSION—LONELINESS AND SOCIAL ISOLATION: THE LANGUAGE(S) OF LONELINESS

**DOI:** 10.1093/geroni/igz038.1365

**Published:** 2019-11-08

**Authors:** Christina Victor, Kimberley Smith

**Affiliations:** 1 Brunel University London, Uxbridge, Middlesex, United Kingdom; 2 University of Surrey, Guildford, Surrey, Canada

## Abstract

We offer a novel perspective on the burgeoning literature focused on loneliness later life by examining the language(s) used to describe, define and depict loneliness. We have an extensive body of work describing the prevalence of , ‘vulnerability factors’ and consequences of loneliness in later life. These activities start with pre-defined concepts of what loneliness is and often use scales and questions which may/may not use the term loneliness. How well does the contemporary language of loneliness used in research, policy, practice and the media really capture the depth and complexity of what people are experiencing? Do the terms and words use in our measurement scales and quantitative research resonate with this vocabulary? In qualitative research interviews how do older adults talk (or avoid talking) about loneliness? How does the media talk about loneliness and what images does this convey about later life? We will address these three issues in our seminar. Using data from qualitative interviews undertaken as part of a mixed methods study of temporal variations in loneliness, Thomas uncovers the strategies participants used to talk or avoid talking about loneliness. Victor uses qualitative data from 12,000 adults aged 60+ collected as part of the BBC loneliness experiment to examine the terms used to describe loneliness and to identify both the opposite of loneliness and the positive aspects of loneliness. Sullivan exposes how loneliness is constructed in print and digital media over a 10-year period in the UK and Canada and its role in framing the loneliness problem.

